# Lineage-specific tissue distribution and high prevalence of haemosporidian parasites in hooded crows (*Corvus cornix*) from northwestern Italy

**DOI:** 10.3389/fvets.2026.1724903

**Published:** 2026-04-29

**Authors:** Laura Starvaggi Cucuzza, Paola Pregel, Anton David Pérez-Rodríguez, Enrico Bollo, Frine Eleonora Scaglione

**Affiliations:** 1 Dipartimento di Scienze Veterinarie, Università degli Studi di Torino, Torino, Italy; 2 Department of Zoology and Entomology, University of the Free State, Bloemfontein, South Africa

**Keywords:** avian malaria, *Corvus cornix*, *Haemoproteus* spp., hooded crows, *Leucocytozoon* spp., multiple infections, *Plasmodium* spp.

## Abstract

Haemosporidian parasites of the genera *Plasmodium*, *Haemoproteus*, and *Leucocytozoon* are widespread vector-borne pathogens of birds, yet their tissue distribution and lineage diversity remain poorly understood in many host species. We investigated haemosporidian infections in hooded crows (*Corvus cornix*) from Northwestern Italy using a multi-organ molecular approach. Forty-seven individuals collected between 2010 and 2011 were screened by nested PCR targeting the mitochondrial cytochrome b gene, followed by sequencing. Overall infection prevalence was 97.9%, with *Leucocytozoon* spp. detected in all infected individuals, followed by *Plasmodium* spp. (52.2%) and *Haemoproteus* spp. (17.4%). Five *Leucocytozoon* lineages were identified, with lineage COCOR09 being dominant and widely distributed across organs (heart, lung, liver, kidney, spleen skeletal muscle and brain), while other lineages showed more restricted tissue patterns. Sequencing also revealed the *Haemoproteus* lineage CXPIP27 and three globally distributed *Plasmodium* lineages (LINN1, GRW06, and SGS1) detected for the first time in this host species. Mixed infections were frequent, occurring in over two-thirds of infected birds. These findings demonstrate extensive tissue dissemination, lineage-specific organ tropism, and high infection complexity in hooded crows, underscoring the importance of multi-organ, lineage-level approaches for understanding the ecology and epidemiology of avian malaria parasites.

## 1 Introduction

Haemosporidian parasites of the genera *Plasmodium*, *Haemoproteus*, and *Leucocytozoon* are vector-borne blood parasites responsible for avian malaria. These parasites exhibit complex heteroxenous life cycles involving avian vertebrate hosts and dipteran insect vectors, and vary markedly in their developmental strategies, vector specificity, and pathogenic potential (1–5). Such biological differences strongly influence host–parasite interactions, transmission dynamics, parasite detection, and disease outcomes.

*Plasmodium* spp., transmitted by mosquitoes (*Culicidae*), undergo both exoerythrocytic and erythrocytic merogony in the avian host (6). After an initial phase of tissue replication, merozoites invade erythrocytes, where repeated schizogonic cycles occur and are often associated with clinical disease (7). In contrast, *Haemoproteus* and *Leucocytozoon* spp. lack erythrocytic schizogony; their asexual replication is restricted to tissue stages, while only gametocytes circulate in peripheral blood (3, 8, 9). *Haemoproteus* spp. are transmitted by biting midges or louse flies, whereas *Leucocytozoon* spp., transmitted mainly by black flies, are characterized by extensive exoerythrocytic development with megaloschizont formation in internal organs such as the liver, spleen, and kidneys (3, 10, 11).

These biological differences have important implications for parasite detection and classification and underpin the widespread use of molecular approaches for haemosporidian lineage identification (8, 12–17).

Avian malaria can severely affect host fitness, causing organ hypertrophy, weight loss, reduced reproductive success, and mortality (18, 19). Clinically, infected birds may exhibit anemia, lethargy, reduced body condition, impaired flight performance, and immunosuppression, while severe infections can lead to multi-organ dysfunction and death, particularly in immunologically naïve hosts (3–5, 20). The disease has also been implicated in population declines, particularly in island ecosystems, with one of the most dramatic examples documented in Hawaii, where the introduction of *Plasmodium relictum* contributed to the extinction of at least seven species of honeycreepers and to severe range contractions in the remaining endemic forest birds (21–24).

Haemosporidian parasites occur worldwide, except in Antarctica, and exhibit high genetic diversity, with infection prevalence varying widely among avian taxa (3, 25–28). This variation likely reflects differences in host immune responses (29, 30) as well as ecological and behavioral factors such as habitat preference, life-history traits, and vector exposure (31–34).

The bird family Corvidae (Passeriformes, Aves), is a cosmopolitan groupcomprising more than 120 species, including crows (*Corvus* spp.), jays (e.g., *Aphelocoma*, *Calocitta*, *Cyanocitta*, *Garrulus* spp.), and magpies (*Pica*, *Cyanopica* spp.). Molecular investigations have revealed a high diversity of haemosporidian lineages in this family, with over 150 lineages recorded to date, including approximately 90 in the genus *Corvus* (12, 14–17, 20, 35–37). While most *Leucocytozoon* species show host specificity at the family or order level (3), *Leucocytozoon berestneffi* and *L. sakharoffi* are considered the most relevant haemosporidian parasites infecting corvids, whereas no *Plasmodium* species appears to be exclusive to the Corvidae family.

The hooded crow (*Corvus cornix* Linnaeus, 1758) is a highly adaptable omnivorous species widely distributed across Italy, inhabiting a range of natural and anthropogenic environments. Its increasing sedentariness over recent decades, particularly in anthropogenic environments (38), may enhance exposure to local vectors and haemosporidian parasites, as reduced movement increases the time available for contact with local vector populations and locally circulating haemoparasites. In contrast, migratory birds, owing to their extensive movements, may experience less continuous exposure to any single vector community, despite encountering more heterogeneous parasite assemblages (39).

Previous studies have demonstrated that avian malaria parasites are not confined to peripheral blood but may be widely distributed across host organs, emphasizing the importance of multi-organ sampling for accurate parasite detection (40). In corvids, post-mortem investigations combining molecular and histopathological approaches have revealed haemosporidian DNA in multiple tissues within the same individual (20), suggesting systemic dissemination beyond peripheral blood. Similar molecular studies conducted in corvids from other European populations have demonstrated a high diversity of *Plasmodium*, *Haemoproteus*, and *Leucocytozoon* lineages, as well as frequent co-infections (35, 37). The application of nested PCR protocols targeting mitochondrial cytochrome b has significantly improved parasite detection sensitivity compared with traditional microscopy (13), enabling lineage discrimination and phylogenetic inference (41). However, in the study by Scaglione et al. (20), lineage-level identification was not performed, limiting comparisons with the growing body of molecular epidemiological data available for corvid hosts. The aim of the present study is therefore to characterize haemosporidian infections in hooded crows by identifying the specific parasite lineages present in multiple organs. By applying molecular sequencing approaches, we seek to clarify whether infections involve single or multiple lineages within individual hosts, thereby improving our understanding of parasite diversity, tissue distribution, and infection patterns in this widespread corvid species.

## 2 Materials and methods

### 2.1 Study area and sample collection

**Figure 1:**
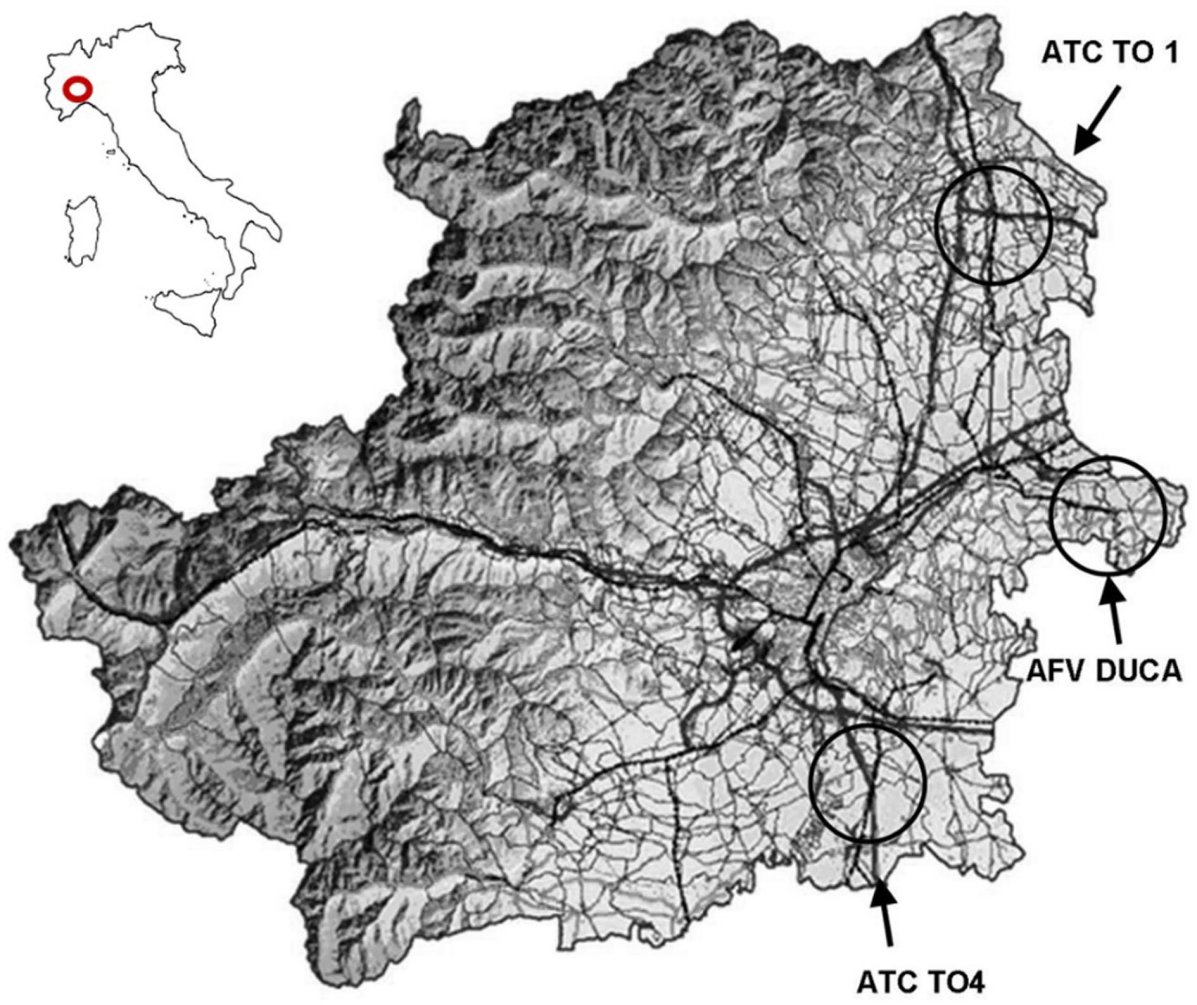
Map of Turin province (Piedmont, Italy), indicating the districts enrolled in the study. Reproduced with permission from (20).

A total of 47 hooded crows (*Corvus cornix*; 27 males and 20 females) were collected between 2010 and 2011 within a regional wildlife control program in the Province of Turin, Northwestern Italy (D.G.R. no. 74-6702 [08/03/2007] and subsequent amendments). Sampling was conducted in three districts (ATC TO1, ATC TO4, and AFV DUCA) (Figure 1), and carcasses were submitted to the Pathology Section of the Department of Veterinary Sciences, University of Turin.

Birds were captured using Larsen cage traps and euthanized with CO₂ by trained personnel in accordance with national legislation and ethical guidelines (DL no. 157, 02/11/1992). A complete necropsy was performed on each individual, and samples of heart, lung, liver, kidney, spleen, skeletal muscle, and brain were collected and stored at −20 °C until molecular analyses.

### 2.2 Parasite detection and sequencing

Genomic DNA was extracted from sampled organs using a commercial DNA isolation kit (Macherey-Nagel, Düren, Germany) according to the manufacturer’s protocol. Haemosporidian parasites (*Plasmodium*, *Haemoproteus*, and *Leucocytozoon* spp.) were detected using a nested polymerase chain reaction (nested PCR) protocol targeting a fragment of the mitochondrial cytochrome *b* gene, following the method described by Hellgren et al. (13), modified as already reported by (20, 42, 43).

In detail: the first PCR amplification was performed using the primers HaemNFl (5′-CATATATTAAGAGAAITATGGAG-3′) and HaemNR3 (5′-ATAGAAAGATAAGAAATACCATTC-3′), which amplify mtDNA from *Plasmodium*, *Haemoproteus*, and *Leucocytozoon* spp. Reactions were carried out in a final volume of 25 μL containing 2 μL of DNA template, 1.5 μM of each primer, and 12.5 μL of HotStarTaq® Master Mix (Qiagen, Hilden, Germany). Amplification was performed after an initial hot-start activation at 95 °C for 15 min, followed by 20 cycles of denaturation at 94 °C for 30 s, annealing at 55 °C for 30 s, and extension at 72 °C for 45 s, with a final extension step at 72 °C for 10 min.

The second PCR step was conducted using 4 μL of the first-round product as template. For *Plasmodium*/*Haemoproteus* spp., primers HaemF (5′-TGGTGCTTTCGATATATGCATG-3′) and HaemR2 (5′-GCATTATCTGGATGTGATAATGGT-3′) were used, whereas *Leucocytozoon* spp. were amplified using primers HaemFL (5′-ATGGT GTTTTAGATACTTACATT-3′) and HaemR2L (5′-CATTATCTGG ATGAGATAATGGIGC-3′). The thermal profile was identical to that of the first PCR step, except for an increased number of cycles (35 cycles). Positive controls consisted of DNA from individuals with known haemosporidian infections, and negative controls were included in each PCR run.

Amplification products were visualized by agarose gel electrophoresis. PCR-positive samples were sequenced on both strands by Macrogen Europe (Amsterdam, Netherlands) using second-round PCR primers. Sequences were manually edited using BioEdit v7.0.5.3 (44) and compared with reference sequences in GenBank using BLAST. Mixed infections were identified based on the presence of double peaks in electropherograms (45). Novel haplotypes were named according to the MalAvi database nomenclature (12) and deposited in GenBank in March 2014 (KJ128987, KJ128988, KJ128989, KJ128990, and KJ128991).

### 2.3 Statistical analysis

Statistical analyses were performed using GraphPad InStat (v3.05). Associations between parasite lineages and organ-specific detection were evaluated using Fisher’s exact test or the Chi-squared (*χ*^2^) test. Statistical significance was set at *p* < 0.05.

## 3 Results

### 3.1 Infection prevalence and parasite genera distribution

Overall, 46 out of 47 hooded crows (97.9%) tested positive for haemosporidian infection, while only one individual (2.1%) was negative. *Leucocytozoon* spp. was the most frequently detected genus, occurring in 100% of PCR-positive birds (*n* = 46), followed by *Plasmodium* spp. (52.2%, *n* = 24) and *Haemoproteus* spp. (17.4%, *n* = 8). All PCR-positive organs were sequenced to discriminate between *Haemoproteus* and *Plasmodium* lineages; however, in a subset of samples, sequencing results remained ambiguous despite repeated attempts. Consequently, lineage-level statistical analyses were restricted to individuals for which complete sequence data were available from all PCR-positive organs, corresponding to 33 of 46 birds infected with *Leucocytozoon* spp. and 24 of 28 birds infected with *Haemoproteus/Plasmodium* spp., calculating the detection rate as the ratio between the number of positive organs/the total number of organs.

### 3.2 *Leucocytozoon* lineage diversity, organ distribution, and phylogeny

**Figure 2:**
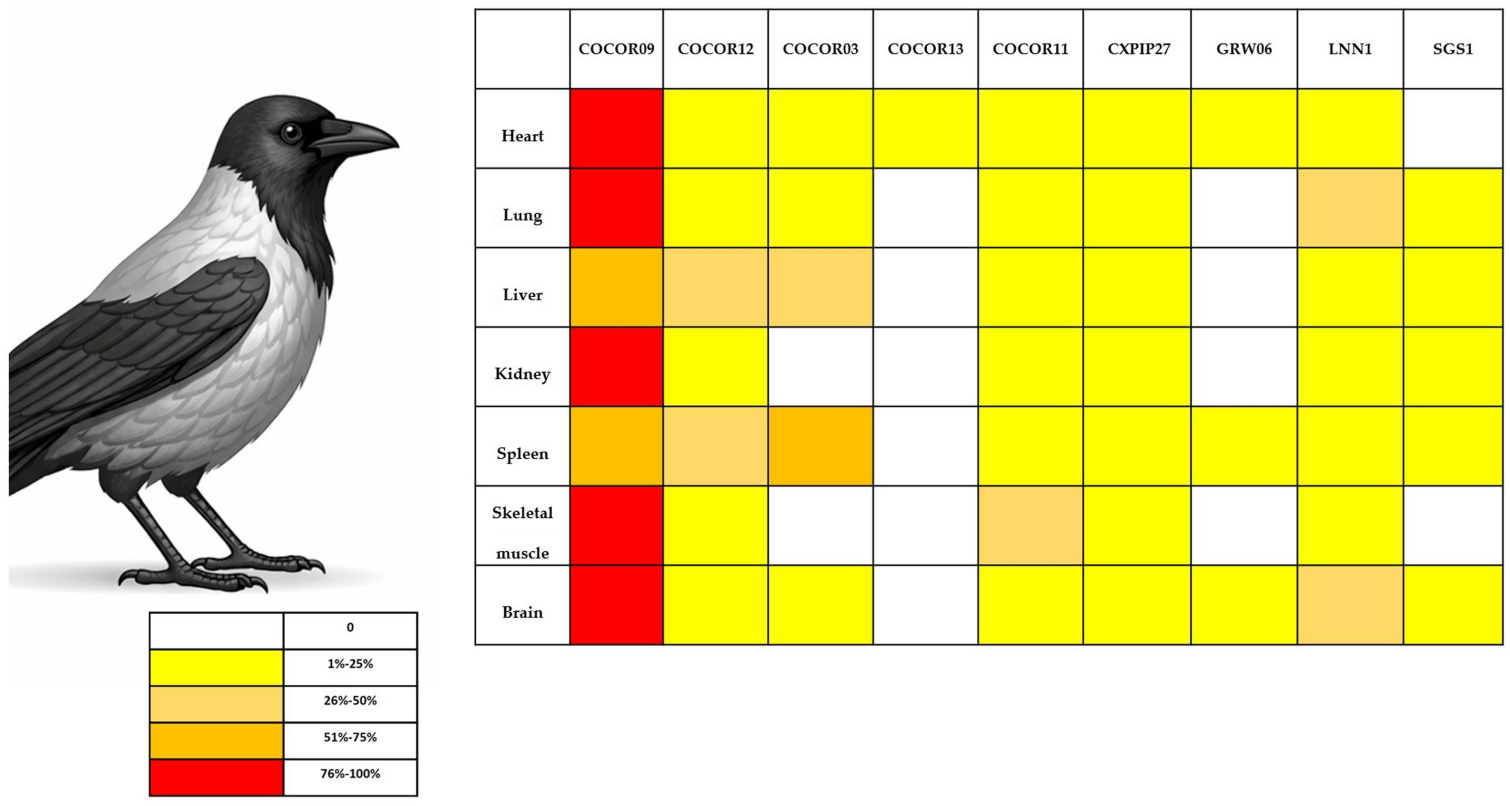
Organ-specific prevalence of *Leucocytozoon* lineages detected in hooded crows (*Corvus cornix*). The figure shows the percentage of examined organs testing positive for each *Leucocytozoon* lineage, as determined by nested PCR. Each bar represents the proportion of organs positive for a given lineage relative to the total number of organs analyzed for that lineage. Lineage COCOR09 was detected in all infected individuals and exhibited consistently high prevalence across all organs, whereas other lineages showed lower and more variable organ-specific detection rates. Lineage COCOR13 was detected exclusively in heart tissue, indicating a highly restricted tissue distribution. Percentages refer to organ-level prevalence, not individual-level infection status.

Five *Leucocytozoon* lineages—COCOR03, COCOR09, COCOR11, COCOR12, and COCOR13—were identified (Table 1). Marked differences in organ-specific prevalence were observed among lineages (Figure 2, Supplementary File 1). Lineage COCOR09 was detected in 100% of infected hooded crows and showed consistently high prevalence across all examined organs, ranging from 91.5 to 96.6%. Intermediate prevalence values were recorded for COCOR03, COCOR12, and COCOR11, with organ-dependent detection rates between 58 and 70%. In contrast, COCOR13 was rare and detected exclusively in the heart, indicating a highly restricted tissue distribution.

**Table 1:** GenBank no., Linage, GenBank sequence, percentage of match and references for the five new different lineages of *Leucocytozoon* identified in the analyzed Hooded crows.

GenBank no.	Lineage	GenBank sequence	% match	Reference
KJ128987	COCOR09	Cc_L2 (MF189963)	100	(37)
		*Corvus corone*, Germany		
		*Corvus corone* 6 (AB741497)	100	
KJ128989	COCOR12	Cc_L5 (MF189966)	99.79	(37)
		*Corvus corone*, Germany		
		Cc_L7 (MF189968)	99.79	(37)
		*Corvus corone*, Germany		
KJ128990	COCOR03	Cc_L3 (MF189964)	100	(37)
		*Corvus corone*, Germany		
KJ128991	COCOR13	Cc_L1 (MF189962)	100	(37)
		*Corvus corone*, Germany		
KJ128988	COCOR11	Cc_L2 (MF189963)	100	(37)
		*Corvus corone*, Germany		

Significant associations among *Leucocytozoon* lineages and positivity in crows were detected (*p* < 0.0001), as well as a strong association between lineage identity and organ tropism (*p* < 0.0001). In addition, the number of lineages infecting an individual was positively correlated with the number of parasitized organs (*p* < 0.05) (Table 2). Single-lineage infections were most common in heart and kidney, whereas double-lineage infections predominated in lung, liver, and spleen. Absence of infection and triple-lineage infections were comparatively rare across all organs.

**Table 2:** Distribution of the number of *Leucocytozoon* lineages detected per organ.

Number of lineage of *Leucocytozoon* per organ	Heart	Lung	Liver	Kidney	Spleen	Skeletal muscle	Brain
0	2	1	2	3	4	3	3
1	20	13	11	20	3	15	16
2	10	19	19	10	25	14	14
3	1	0	1	0	1	1	0

The table shows the frequency of samples with 0, 1, 2, or 3 *Leucocytozoon* lineages identified in each examined organ.

### 3.3 *Haemoproteus* and *Plasmodium* lineages

Sequencing of *Haemoproteus/Plasmodium*-positive samples revealed the presence of multiple lineages. During the 2010–2011 sampling period, we identified the *Haemoproteus* lineage CXPIP27 (GenBank accession KJ128983), reported in 2014 from northwestern Iberia (Table 3). In addition, three *Plasmodium* lineages—LINN1 (KJ128984), GRW06 (KJ128985), and SGS1 (KJ128986)—were detected in hooded crows, representing the first records of these MalAvi lineages in this host species (Table 3).

**Table 3:** GenBank no., Linage, GenBank sequence, percentage of match and references for the five new different lineages of *Haemoproteus* and *Plasmodium* identified in the analyzed Hooded crows.

GenBank no.	Lineage	GenBank sequence	% match	Reference
KJ128983	CXPIP27	H360 (KJ488905)	100	(49)
		*Corvus corone*, Spain		
KJ128985	GRW06	GRW06 (DQ368381)	100	(52)
		*Acrocephalus arundinaceus*		
		GRW06, (DQ368381)	100	(35)
		*Corvus corax*, Bulgaria		
		H012 (KJ488566)	100	(49)
		*Cettia cetti*, Transcaucasia		
KJ128984	LNN1	LINN1 (MF817787)	100	(55)
		*Turdus migratorius*, Michigan (United States)		
		*Plasmodium matutinum* isolate 9/16R (KY287235)	100	(53)
		*Luscinia luscinia*, Lithuania		
		WWT002 (KX867108)	100	(51)
		*Baeolophus bicolor*, Mississippi, (United States)		
		H144 (KJ488698)	100	(49)
		*Cyanistes caeruleus*, western Greater Caucasus		
		*Turdidae*, northwest Iberia, western Greater Caucasus and Transcaucasia		
KJ128986	SGS1	*Plasmodium relictum* strain Cc_Pp_P (MF189958)	100	(37)
		*Corvus corone*, Germany		
		*Plasmodium relictum* strain H011 (KJ488565)	100	(49)
		*Garrulus glandarius*, western Greater Caucasus and Transcaucasia		
		*Cyanopica cooki*, northwest Iberia		

Similar to *Leucocytozoon*, significant associations among *Plasmodium* lineages and crows (*p* = 0.001) were observed. *Haemoproteus/Plasmodium* spp. was detected in a limited number of organs, with the spleen being the most frequently positive organ. Single-lineage infections of *Haemoproteus* (1 lineage) were detected only sporadically and at low frequency, occurring in a small number of samples in the heart (5), liver (4), skeletal muscle (4), kidney (2), lung (1), spleen (1), and brain (1). No cases of multiple infections (2 or 3 lineages) were observed in any organ (Table 4, Supplementary File 2).

**Table 4:** Distribution of the number of *Haemoproteus* lineages detected per organ.

Number of lineage of *Haemoproteus* per organ	Heart	Lung	Liver	Kidney	Spleen	Skeletal muscle	Brain
0	28	32	29	31	32	29	32
1	5	1	4	2	1	4	1
2	0	0	0	0	0	0	0
3	0	0	0	0	0	0	0

The table shows the frequency of samples with 0, 1, 2, or 3 *Hemoproteus* lineages identified in each examined organ.

The distribution of *Plasmodium* lineages per organ shows that most samples were negative (0 lineages), particularly in the heart, liver, and kidney (29 negative samples each). Single-lineage infections (1 lineage) were more frequently detected in certain organs. The spleen (13 cases), lung (12 cases), and brain (12 cases) exhibited the highest number of single-lineage infections, suggesting a relatively greater presence of *Plasmodium* in these tissues. Skeletal muscle showed intermediate values (6 cases), whereas heart, liver, and kidney had fewer positive samples (4 cases each). No multiple infections (2 or 3 lineages) were observed in any organ. Overall, the results indicate that *Plasmodium* infections were generally low in frequency and restricted to single-lineage detections, with a higher occurrence in spleen, lung, and brain compared to other organs (Table 5, Supplementary File 3).

**Table 5:** Distribution of the number of *Plasmodium* lineages detected per organ.

Number of lineage of *Plasmodium* per organ	Heart	Lung	Liver	Kidney	Spleen	Skeletal muscle	Brain
0	29	21	29	29	20	27	21
1	4	12	4	4	13	6	12
2	0	0	0	0	0	0	0
3	0	0	0	0	0	0	0

The table shows the frequency of samples with 0, 1, 2, or 3 *Plasmodium* lineages identified in each examined organ.

### 3.4 Mixed infections and organ-level patterns

Mixed infections involving two or more haemosporidian lineages were detected in 31 of 46 infected hooded crows (67.4%) (Table 6). Co-infections involving multiple parasite genera were observed in 18 individuals (58.1%). Intra-generic co-infections predominated (93.5%), most frequently involving *Leucocytozoon* lineages, followed by *Plasmodium* lineages. Among intergeneric infections, the most common combination was *Leucocytozoon* + *Plasmodium* (*n* = 13), whereas coinfections involving *Haemoproteus* were rare. Triple infections involving all three genera were detected in three individuals.

**Table 6:** Distribution of haemosporidian parasite genera detected across examined organs in hooded crows (*n* = 31).

Organs	Number of haemoparasites genera				Total
	3	2	1	0	
Heart	1	2	1	0	
Lung	0	10	19	2	
Liver	0	6	23	2	
Kidney	0	5	22	4	
Spleen	1	10	16	4	
Skeletal muscle	0	8	20	3	
CNS	0	8	21	2	
Total	2	53	142	20	217
Percentage	0.9%	24.4%	65.4%	9.2%	

More than half of the birds infected with *Leucocytozoon* (53.3%) harbored at least one additional lineage. Notably, 41.9% of co-infected individuals carried four distinct lineages, and the most heavily parasitized bird harbored up to seven lineages, with all three genera detected in both heart and spleen. When data from all organs and genera were pooled, 9.2% of organs were negative for haemosporidians, whereas only 0.9% were simultaneously positive for all three genera. Most organs (65.4%) were positive for a single parasite genus.

To clarify the interpretation of organ-level patterns, Table 6 summarizes the number of haemosporidian genera detected per individual organ, rather than per host, thereby illustrating the distribution of single- and multi-genus detections across tissues.

## 4 Discussion

This study provides a lineage-level characterization of haemosporidian infections in hooded crows (*Corvus cornix*) from Northwestern Italy, revealing a very high prevalence of infection and substantial parasite diversity. The overall infection rate observed is consistent with previous molecular studies in corvids and confirms that this taxonomic group represents a highly permissive host assemblage for haemosporidian parasites (14–17, 35, 37). In particular, the dominance of *Leucocytozoon* spp., detected in all infected individuals, reinforces the strong association between corvids and this parasite genus.

The predominance of *Leucocytozoon* infections likely reflects vector-related factors, as black flies (*Simuliidae*) may exhibit relatively narrow host preferences and high transmission efficiency within specific avian groups (3). In contrast, mosquitoes transmitting *Plasmodium* spp. are generally more opportunistic feeders, potentially reducing transmission efficiency in corvids. These differences in vector ecology may explain the markedly higher prevalence of *Leucocytozoon* compared to other haemosporidian genera in this and other corvid populations (46).

The multi-organ sampling approach adopted here proved critical for capturing the full extent of infection. Haemosporidian parasites were widely distributed across host tissues, confirming that infections are systemic rather than restricted to peripheral blood (3, 40). Moreover, the strong association observed between lineage identity and organ tropism suggests that tissue distribution is not random but reflects lineage-specific biological traits, potentially linked to differences in parasite development or host immune interactions (47).

Among *Leucocytozoon* lineages, COCOR09 was clearly dominant, being detected in all infected individuals and across nearly all organs. Its widespread occurrence and high organ-level prevalence suggest a well-established host–parasite relationship, since the lack of pathological signs may indicate a stable interaction and host tolerance. Furthermore, such high prevalence values imply that the parasite is widely circulating within the host population (3, 54)). In contrast, other lineages showed more restricted distributions, particularly COCOR13, which was detected only in heart tissue. Such heterogeneity supports the idea that different *Leucocytozoon* lineages occupy distinct ecological niches within the host, facilitating their coexistence (48).

Lineages COCOR09, COCOR03, COCOR13, and COCOR11 showed 100% sequence identity with lineages previously reported from carrion crows (*Corvus corone*) in Germany, and COCOR09 was also identical to a sequence detected in *C. corone* from Japan (Table 1). No sequence in GenBank or the MalAvi database matched COCOR12 with complete identity, although this lineage showed high similarity to sequences isolated from carrion crows in Germany (37).

The detection of the *Haemoproteus* lineage CXPIP27 prior to its first formal description (49) highlights the value of retrospective molecular analyses in assessing whether a parasite was already present in geographic areas that had not yet been investigated. Similarly, the identification of the *Plasmodium* lineages LINN1, GRW06, and SGS1—all known generalists—in hooded crows supports the notion that *Plasmodium* parasites frequently cross host-species boundaries via shared vectors (50). Moreover, the LINN1 lineage has been reported in passerine hosts and showed 100% identity with a sequence obtained from *Luscinia luscinia* in Lithuania (51). The GRW06 and SGS1 lineages, corresponding to widely distributed MalAvi lineages, have been frequently reported across multiple avian taxa worldwide.

## 5 Conclusion

In conclusion, hooded crows from Northwestern Italy host a diverse and complex assemblage of haemosporidian parasites characterized by extensive tissue dissemination, lineage-specific organ tropism, and frequent mixed infections. The predominance of intra-generic co-infections, especially among *Leucocytozoon* lineages, suggests repeated exposure to infective vectors and the ability of multiple lineages to persist within the same host. Such complex infection patterns may influence parasite competition, host immune responses, and transmission dynamics, although the direction and magnitude of these effects remain to be clarified. These findings highlight the potential role of corvids as important reservoirs for avian malaria parasites and underscore the value of multi-organ, lineage-level approaches for understanding the ecology and epidemiology of haemosporidian infections in natural bird populations.

## Data Availability

The datasets presented in this study can be found in online repositories. The names of the repository/repositories and accession number(s) can be found in the article/Supplementary material.
