# Supplementary material for: Lineage-specific tissue distribution and high prevalence of haemosporidian parasites in hooded crows (Corvus cornix) from northwestern Italy
Source: Front Vet Sci. 2026 Apr 29;13:1724903. doi: 10.3389/fvets.2026.1724903 (PMC13169154; doi:10.3389/fvets.2026.1724903)
Supplement: Supplementary file 1 [file Table_1.docx]

| Case # | *Leucocytozoon* spp. | | | | | | | *Number of lineages per organ* | | | | | | |
| --- | --- | --- | --- | --- | --- | --- | --- | --- | --- | --- | --- | --- | --- | --- |
|  | Heart | Lung | Liver | Kidney | Spleen | Skeletal muscle | Brain | Heart | Lung | Liver | Kidney | Spleen | Skeletal muscle | Brain |
| 3 | COCOR09 | COCOR09 | COCOR09 | COCOR09 | COCOR09 | COCOR09 | COCOR09 | 1 | 2 | 2 | 1 | 1 | 1 | 1 |
|  |  | COCOR12 | COCOR12 |  |  |  |  |  |  |  |  |  |  |  |
| 4 | COCOR09 | COCOR09 | COCOR09 | neg | COCOR09 | COCOR09 | COCOR09 | 1 | 1 | 1 |  | 2 | 2 | 1 |
|  |  |  |  |  | COCOR03 | COCOR11 |  |  |  |  |  |  |  |  |
| 5 | COCOR09 | COCOR12 | COCOR09 | COCOR09 | COCOR09 | COCOR09 | COCOR09 | 1 | 2 | 2 | 2 | 2 | 2 | 1 |
|  |  | COCOR09 | COCOR11 | COCOR11 | COCOR03 | COCOR11 |  |  |  |  |  |  |  |  |
| 7 | COCOR09 | COCOR09 | COCOR12 | COCOR09 | neg | COCOR09 | COCOR09 | 2 | 2 | 2 | 1 |  | 2 | 2 |
|  | COCOR12 | COCOR12 | COCOR11 |  |  | COCOR12 | COCOR12 |  |  |  |  |  |  |  |
| 10 | COCOR09 | COCOR09 | COCOR09 | COCOR09 | COCOR09 | COCOR09 | COCOR09 | 1 | 2 | 1 | 2 | 2 | 2 | 2 |
|  |  | COCOR03 |  | COCOR11 | COCOR03 | COCOR11 | COCOR11 |  |  |  |  |  |  |  |
| 15 | COCOR09 | COCOR12 | COCOR12 | COCOR09 | neg | COCOR12 | COCOR12 | 2 | 1 | 1 | 2 |  | 2 | 1 |
|  | COCOR12 |  |  | COCOR12 |  | COCOR09 |  |  |  |  |  |  |  |  |
| 17 | COCOR11 | COCOR11 | COCOR03 | COCOR11 | COCOR09 | COCOR11 | COCOR11 | 1 | 1 | 2 | 1 | 2 | 1 | 1 |
|  |  |  | COCOR11 |  | COCOR12 |  |  |  |  |  |  |  |  |  |
| 18 | COCOR09 | COCOR09 | COCOR03 | COCOR09 | COCOR09 | COCOR09 | neg | 1 | 2 | 1 | 2 | 2 | 1 |  |
|  |  | COCOR03 |  | COCOR11 | COCOR03 |  |  |  |  |  |  |  |  |  |
| 19 | COCOR09 | COCOR09 | COCOR09 | COCOR09 | COCOR09 | COCOR09 | COCOR09 | 1 | 1 | 2 | 1 | 3 | 1 | 1 |
|  |  |  |  |  | COCOR11 |  |  |  |  |  |  |  |  |  |
|  |  |  | COCOR12 |  | COCOR03 |  |  |  |  |  |  |  |  |  |
| 20 | COCOR09 | COCOR09 | COCOR12 | COCOR09 | COCOR09 | neg | COCOR09 | 2 | 2 | 2 | 1 | 2 |  | 2 |
|  | COCOR11 | COCOR03 | COCOR03 |  | COCOR03 |  | COCOR12 |  |  |  |  |  |  |  |
| 21 | COCOR09 | COCOR09 | COCOR09 | COCOR09 | COCOR09 | COCOR09 | COCOR09 | 2 | 2 | 2 | 2 | 2 | 2 | 2 |
|  | COCOR11 | COCOR12 | COCOR03 | COCOR12 | COCOR03 | COCOR11 | COCOR03 |  |  |  |  |  |  |  |
| 22 | COCOR09 | COCOR09 | COCOR09 | COCOR09 | COCOR09 | COCOR09 | COCOR09 | 1 | 2 | 1 | 1 | 2 | 1 | 2 |
|  |  | COCOR03 |  |  | COCOR03 |  | COCOR12 |  |  |  |  |  |  |  |
| 23 | neg | neg | neg | neg | neg | neg | neg |  |  |  |  |  |  |  |
|  |  |  |  |  |  |  |  |  |  |  |  |  |  |  |
| 24 | COCOR09 | COCOR09 | COCOR09 | COCOR09 | COCOR09 | COCOR09 | COCOR12 | 1 | 2 | 2 | 1 | 2 | 1 | 2 |
|  |  | COCOR03 | COCOR12 |  | COCOR03 |  | COCOR03 |  |  |  |  |  |  |  |
| 26 | COCOR12 | COCOR09 | COCOR09 | COCOR09 | COCOR09 | COCOR09 | COCOR09 | 2 | 2 | 2 | 2 | 2 | 2 | 2 |
|  | COCOR11 | COCOR11 | COCOR03 | COCOR11 | COCOR03 | COCOR11 | COCOR11 |  |  |  |  |  |  |  |
| 27 | COCOR09 | COCOR09 | COCOR09 | COCOR09 | COCOR09 | COCOR09 | COCOR09 | 2 | 2 | 1 | 1 | 2 | 1 | 1 |
|  | COCOR03 | COCOR11 |  |  | COCOR03 |  |  |  |  |  |  |  |  |  |
| 28 | COCOR09 | COCOR09 | COCOR09 | COCOR09 | COCOR09 | COCOR03 | neg | 1 | 2 | 2 | 1 | 2 | 2 |  |
|  |  | COCOR03 | COCOR03 |  | COCOR03 | COCOR11 |  |  |  |  |  |  |  |  |
| 29 | COCOR09 | COCOR09 | COCOR09 | COCOR09 | COCOR09 | neg | COCOR09 | 1 | 1 | 2 | 1 | 1 |  | 2 |
|  |  |  | COCOR03 |  |  |  | COCOR12 |  |  |  |  |  |  |  |
| 30 | COCOR09 | COCOR09 | COCOR09 | COCOR09 | COCOR09 | COCOR09 | COCOR09 | 1 | 1 | 1 | 1 | 2 | 1 | 1 |
|  |  |  |  |  | COCOR03 |  |  |  |  |  |  |  |  |  |
| 31 | COCOR09 | COCOR09 | COCOR09 | COCOR09 | COCOR09 | COCOR09 | COCOR09 | 1 | 1 | 1 | 1 | 2 | 2 | 2 |
|  |  |  |  |  | COCOR03 | COCOR12 | COCOR03 |  |  |  |  |  |  |  |
| 32 | COCOR09 | COCOR09 | neg | COCOR09 | COCOR09 | COCOR09 | COCOR09 | 1 | 1 |  | 1 | 1 | 1 | 1 |
|  |  |  |  |  |  |  |  |  |  |  |  |  |  |  |
| 33 | COCOR09 | COCOR09 | COCOR09 | COCOR09 | COCOR12 | COCOR09 | COCOR09 | 1 | 2 | 2 | 2 | 2 | 1 | 1 |
|  |  | COCOR03 | COCOR03 | COCOR11 | COCOR03 |  |  |  |  |  |  |  |  |  |
| 34 | COCOR09 | COCOR09 | COCOR12 | COCOR09 | COCOR12 | COCOR09 | COCOR09 | 1 | 1 | 2 | 1 | 2 | 1 | 1 |
|  |  |  | COCOR03 |  | COCOR03 |  |  |  |  |  |  |  |  |  |
| 35 | COCOR09 | COCOR09 | COCOR09 | COCOR09 | COCOR09 | COCOR09 | COCOR09 | 3 | 2 | 3 | 1 | 2 | 2 | 1 |
|  | COCOR12 |  | COCOR12 |  |  |  |  |  |  |  |  |  |  |  |
|  | COCOR13 | COCOR12 | COCOR11 |  | COCOR12 | COCOR12 |  |  |  |  |  |  |  |  |
| 36 | COCOR09 | COCOR09 | COCOR09 | COCOR09 | COCOR12 | COCOR09 | COCOR09 | 2 | 1 | 2 | 2 | 2 | 1 | 2 |
|  | COCOR11 |  | COCOR12 | COCOR11 | COCOR11 |  | COCOR03 |  |  |  |  |  |  |  |
| 37 | COCOR09 | COCOR09 | COCOR09 | COCOR09 | COCOR12 | COCOR09 | COCOR09 | 2 | 2 | 2 | 2 | 2 | 2 | 2 |
|  | COCOR11 | COCOR11 | COCOR03 | COCOR11 | COCOR03 | COCOR11 | COCOR03 |  |  |  |  |  |  |  |
| 38 | COCOR09 | COCOR09 | COCOR09 | COCOR09 | COCOR09 | COCOR09 | COCOR09 | 1 | 1 | 2 | 1 | 2 | 3 | 1 |
|  |  |  |  |  |  | COCOR12 |  |  |  |  |  |  |  |  |
|  |  |  | COCOR12 |  | COCOR12 | COCOR11 |  |  |  |  |  |  |  |  |
| 40 | COCOR09 | COCOR09 | COCOR09 | COCOR09 | COCOR09 | COCOR09 | COCOR09 | 1 | 1 | 1 | 1 | 2 | 1 | 1 |
|  |  |  |  |  | COCOR12 |  |  |  |  |  |  |  |  |  |
| 41 | neg | COCOR09 | neg | neg | COCOR12 | neg | neg |  | 1 |  |  | 2 |  |  |
|  |  |  |  |  | COCOR03 |  |  |  |  |  |  |  |  |  |
| 42 | COCOR12 | COCOR09 | COCOR09 | COCOR09 | COCOR09 | COCOR09 | COCOR12 | 1 | 2 | 2 | 2 | 2 | 2 | 2 |
|  |  | COCOR03 | COCOR12 | COCOR03 | COCOR03 | COCOR03 | COCOR11 |  |  |  |  |  |  |  |
| 43 | COCOR09 | COCOR09 | COCOR09 | COCOR09 | COCOR09 | COCOR09 | COCOR09 | 2 | 2 | 2 | 1 | 2 | 2 | 2 |
|  | COCOR03 | COCOR12 | COCOR11 |  | COCOR12 | COCOR12 | COCOR12 |  |  |  |  |  |  |  |
| 44 | neg | COCOR09 | COCOR09 | COCOR09 | neg | COCOR09 | COCOR09 |  | 2 | 1 | 1 |  | 1 | 1 |
|  |  | COCOR12 |  |  |  |  |  |  |  |  |  |  |  |  |
| 45 | COCOR09 | 0 | COCOR09 | COCOR09 | COCOR09 | COCOR09 | COCOR09 | 1 |  | 2 | 1 | 2 | 1 | 1 |
|  |  |  | COCOR03 |  | COCOR13 |  |  |  |  |  |  |  |  |  |
| 46 | COCOR09 | COCOR09 | COCOR09 | neg | neg | COCOR09 | COCOR09 | 2 | 2 | 1 |  |  | 2 | 2 |
|  | COCOR12 | COCOR11 |  |  |  | COCOR11 | COCOR13 |  |  |  |  |  |  |  |

Supplementary file 1: Organ-specific distribution of Leucocytozoon lineages in individual cases. The table reports, for each examined case, the Leucocytozoon lineages detected in each organ (heart, lung, liver, kidney, spleen, skeletal muscle, and brain) together with the corresponding number of lineages per organ. “Neg” indicates absence of detectable infection.
