# Supplementary material for: Lineage-specific tissue distribution and high prevalence of haemosporidian parasites in hooded crows (Corvus cornix) from northwestern Italy
Source: Front Vet Sci. 2026 Apr 29;13:1724903. doi: 10.3389/fvets.2026.1724903 (PMC13169154; doi:10.3389/fvets.2026.1724903)
Supplement: Supplementary file 2 [file Table_2.docx]

| Case # | *Haemoproteus* spp*.* | | | | | | | *Number of lineages per organ* | | | | | | |
| --- | --- | --- | --- | --- | --- | --- | --- | --- | --- | --- | --- | --- | --- | --- |
|  | Heart | Lung | Liver | Kidney | Spleen | Skeletal muscle | Brain | Heart | Lung | Liver | Kidney | Spleen | Skeletal muscle | Brain |
| 1 | neg | neg | neg | neg | neg | neg | neg |  |  |  |  |  |  |  |
| 2 | neg | neg | neg | neg | neg | neg | neg |  |  |  |  |  |  |  |
| 3 | neg | neg | neg | neg | neg | neg | neg |  |  |  |  |  |  |  |
| 4 | neg | neg | neg | neg | neg | neg | neg |  |  |  |  |  |  |  |
| 5 | CXPIP27 | neg | CXPIP27 | neg | CXPIP27 | neg | neg | 1 |  | 1 |  | 1 |  |  |
| 7 | CXPIP27 | neg | neg | CXPIP27 | neg | CXPIP27 | neg | 1 |  |  | 1 |  | 1 |  |
| 8 | neg | neg | neg | neg | neg | neg | neg |  |  |  |  |  |  |  |
| 9 | CXPIP27 | neg | neg | neg | neg | CXPIP27 | CXPIP27 | 1 |  |  |  |  | 1 | 1 |
| 10 | CXPIP27 | neg | CXPIP27 | CXPIP27 | neg | CXPIP27 | neg | 1 |  | 1 | 1 |  | 1 |  |
| 11 | neg | neg | neg | neg | neg | neg | neg |  |  |  |  |  |  |  |
| 12 | neg | neg | neg | neg | neg | neg | neg |  |  |  |  |  |  |  |
| 13 | neg | neg | CXPIP27 | neg | neg | neg | neg |  |  | 1 |  |  |  |  |
| 14 | neg | neg | neg | neg | neg | neg | neg |  |  |  |  |  |  |  |
| 15 | neg | neg | neg | neg | neg | neg | neg |  |  |  |  |  |  |  |
| 16 | neg | neg | neg | neg | neg | neg | neg |  |  |  |  |  |  |  |
| 17 | neg | neg | neg | neg | neg | neg | neg |  |  |  |  |  |  |  |
| 18 | neg | neg | neg | neg | neg | neg | neg |  |  |  |  |  |  |  |
| 21 | neg | neg | neg | neg | neg | neg | neg |  |  |  |  |  |  |  |
| 22 | neg | neg | neg | neg | neg | neg | neg |  |  |  |  |  |  |  |
| 23 | neg | neg | CXPIP27 | neg | neg | neg | neg |  |  | 1 |  |  |  |  |
| 24 | neg | neg | neg | neg | neg | neg | neg |  |  |  |  |  |  |  |
| 25 | neg | neg | neg | neg | neg | neg | neg |  |  |  |  |  |  |  |
| 26 | neg | neg | neg | neg | neg | neg | neg |  |  |  |  |  |  |  |
| 27 | neg | neg | neg | neg | neg | neg | neg |  |  |  |  |  |  |  |
| 28 | neg | neg | neg | neg | neg | neg | neg |  |  |  |  |  |  |  |
| 29 | neg | neg | neg | neg | neg | neg | neg |  |  |  |  |  |  |  |
| 30 | CXPIP27 | CXPIP27 | neg | neg | neg | CXPIP27 | neg | 1 | 1 |  |  |  | 1 |  |
| 31 | neg | neg | neg | neg | neg | neg | neg |  |  |  |  |  |  |  |
| 32 | neg | neg | neg | neg | neg | neg | neg |  |  |  |  |  |  |  |
| 33 | neg | neg | neg | neg | neg | neg | neg |  |  |  |  |  |  |  |
| 34 | neg | neg | neg | neg | neg | neg | neg |  |  |  |  |  |  |  |
| 35 | neg | neg | neg | neg | neg | neg | neg |  |  |  |  |  |  |  |
| 36 | neg | neg | neg | neg | neg | neg | neg |  |  |  |  |  |  |  |
| 37 | neg | neg | neg | neg | neg | neg | neg |  |  |  |  |  |  |  |
| 38 | neg | neg | neg | neg | neg | neg | neg |  |  |  |  |  |  |  |
| 39 | neg | neg | neg | neg | neg | neg | neg |  |  |  |  |  |  |  |
| 40 | neg | neg | neg | neg | neg | neg | neg |  |  |  |  |  |  |  |
| 41 | neg | neg | neg | neg | neg | neg | neg |  |  |  |  |  |  |  |
| 43 | neg | neg | neg | neg | neg | neg | neg |  |  |  |  |  |  |  |
| 44 | neg | neg | neg | neg | neg | neg | neg |  |  |  |  |  |  |  |
| 45 | neg | neg | neg | neg | neg | neg | neg |  |  |  |  |  |  |  |
| 46 | neg | neg | neg | neg | neg | neg | neg |  |  |  |  |  |  |  |
| 47 | neg | neg | neg | neg | neg | neg | neg |  |  |  |  |  |  |  |

Supplementary file 2: Organ-specific distribution of Haemoproteus lineages in individual cases. The table reports, for each examined case, the Haemoproteus lineage detected in each organ (heart, lung, liver, kidney, spleen, skeletal muscle, and brain) and the corresponding number of lineages per organ. “Neg” indicates absence of detectable infection
