# Supplementary material for: Lineage-specific tissue distribution and high prevalence of haemosporidian parasites in hooded crows (Corvus cornix) from northwestern Italy
Source: Front Vet Sci. 2026 Apr 29;13:1724903. doi: 10.3389/fvets.2026.1724903 (PMC13169154; doi:10.3389/fvets.2026.1724903)
Supplement: Supplementary file 3 [file Table_3.docx]

| Case # | *Plasmodium* spp*.* | | | | | | | *Number of lineages per organ* | | | | | | |
| --- | --- | --- | --- | --- | --- | --- | --- | --- | --- | --- | --- | --- | --- | --- |
|  | Heart | Lung | Liver | Kidney | Spleen | Skeletal muscle | Brain | Heart | Lung | Liver | Kidney | Spleen | Skeletal muscle | Brain |
| 1 | neg | neg | neg | neg | SGS1 | neg | neg |  |  |  |  | 1 |  |  |
| 2 | neg | neg | neg | neg | SGS1 | neg | neg |  |  |  |  | 1 |  |  |
| 3 | neg | SGS1 | neg | SGS1 | LINN1 | neg | LINN1 |  | 1 |  | 1 | 1 |  | 1 |
| 4 | neg | LINN1 | LINN1 | neg | LINN1 | neg | neg |  | 1 |  | 1 | 1 |  | 1 |
| 5 | GRW06 | LINN1 | neg | neg | LINN1 | neg | LINN1 | 1 | 1 |  |  | 1 |  | 1 |
| 7 | neg | SGS1 | SGS1 | neg | neg | neg | neg |  | 1 | 1 |  |  |  |  |
| 8 | neg | LINN1 | SGS1 | neg | SGS1 | neg | neg |  | 1 | 1 |  | 1 |  |  |
| 9 | neg | neg | neg | neg | neg | neg | neg |  |  |  |  |  |  |  |
| 10 | neg | LINN1 | neg | neg | SGS1 | neg | LINN1 |  | 1 |  |  | 1 |  | 1 |
| 11 | neg | neg | neg | neg | neg | neg | neg |  |  |  |  |  |  |  |
| 12 | neg | SGS1 | neg | neg | neg | neg | neg |  | 1 |  |  |  |  |  |
| 13 | neg | LINN1 | neg | LINN1 | neg | LINN1 | LINN1 |  | 1 |  | 1 |  | 1 | 1 |
| 14 | neg | neg | neg | neg | neg | neg | neg |  |  |  |  |  |  |  |
| 15 | neg | neg | neg | neg | neg | neg | SGS1 |  |  |  |  |  |  | 1 |
| 16 | neg | neg | neg | neg | neg | neg | neg |  |  |  |  |  |  |  |
| 17 | LINN1 | LINN1 | SGS1 | LINN1 | SGS1 | LINN1 | LINN1 | 1 | 1 | 1 | 1 | 1 | 1 | 1 |
| 18 | LINN1 | LINN1 | neg | neg | LINN1 | LINN1 | LINN1 | 1 | 1 |  |  | 1 | 1 | 1 |
| 21 | neg | neg | neg | neg | SGS1 | neg | neg |  |  |  |  | 1 |  |  |
| 22 | neg | neg | neg | neg | neg | LINN1 | LINN1 |  |  |  |  |  | 1 | 1 |
| 23 | neg | neg | neg | neg | SGS1 | neg | LINN1 |  |  |  |  | 1 |  | 1 |
| 24 | neg | neg | neg | neg | LINN1 | neg | neg |  |  |  |  | 1 |  |  |
| 25 | neg | neg | neg | neg | neg | neg | neg |  |  |  |  |  |  |  |
| 26 | GRW06 | LINN1 | neg | neg | GRW06 | LINN1 | LINN1 | 1 | 1 |  |  | 1 | 1 | 1 |
| 27 | neg | neg | neg | LINN1 | neg | neg | neg |  |  |  |  |  |  |  |
| 28 | neg | LINN1 | LINN1 | neg | LINN1 | LINN1 | neg |  | 1 | 1 |  | 1 | 1 |  |
| 29 | neg | neg | neg | neg | neg | neg | GRW06 |  |  |  |  |  |  | 1 |
| 30 | neg | neg | neg | neg | neg | neg | neg |  |  |  |  |  |  |  |
| 31 | neg | neg | neg | neg | neg | neg | neg |  |  |  |  |  |  |  |
| 32 | neg | neg | neg | neg | neg | neg | neg |  |  |  |  |  |  |  |
| 33 | neg | neg | neg | neg | neg | neg | neg |  |  |  |  |  |  |  |
| 34 | neg | neg | neg | neg | GRW06 | neg | neg |  |  |  |  | 1 |  |  |
| 35 | neg | neg | neg | neg | neg | neg | neg |  |  |  |  |  |  |  |
| 36 | neg | neg | neg | neg | neg | neg | neg |  |  |  |  |  |  |  |
| 37 | neg | neg | neg | neg | neg | neg | neg |  |  |  |  |  |  |  |
| 38 | neg | neg | neg | neg | neg | neg | neg |  |  |  |  |  |  |  |
| 39 | neg | neg | neg | neg | neg | neg | neg |  |  |  |  |  |  |  |
| 40 | neg | neg | neg | neg | neg | neg | neg |  |  |  |  |  |  |  |
| 41 | neg | neg | neg | neg | neg | neg | neg |  |  |  |  |  |  |  |
| 43 | neg | neg | neg | neg | neg | neg | neg |  |  |  |  |  |  |  |
| 44 | neg | neg | neg | neg | neg | neg | neg |  |  |  |  |  |  |  |
| 45 | neg | neg | neg | neg | neg | neg | neg |  |  |  |  |  |  |  |
| 46 | neg | neg | neg | neg | neg | neg | neg |  |  |  |  |  |  |  |
| 47 | neg | neg | neg | neg | neg | neg | neg |  |  |  |  |  |  |  |

Supplementary file 3: Organ-specific distribution of *Plasmodium* lineages in individual cases. The table reports, for each examined case, the Plasmodium lineage detected in each organ (heart, lung, liver, kidney, spleen, skeletal muscle, and brain) and the corresponding number of lineages per organ. “Neg” indicates absence of detectable infection.
